# Multiple Hepatic Hydatid Cysts in an Iraqi Refugee

**DOI:** 10.4269/ajtmh.18-0371

**Published:** 2018-11

**Authors:** Amila Heendeniya, Isaac I. Bogoch

**Affiliations:** 1 Department of Medicine, University of Toronto, Toronto, Ontario, Canada;; 2 Divisions of General Internal Medicine and Infectious Diseases, Toronto General Hospital, University Health Network, Toronto, Ontario, Canada

**Figure 1.**
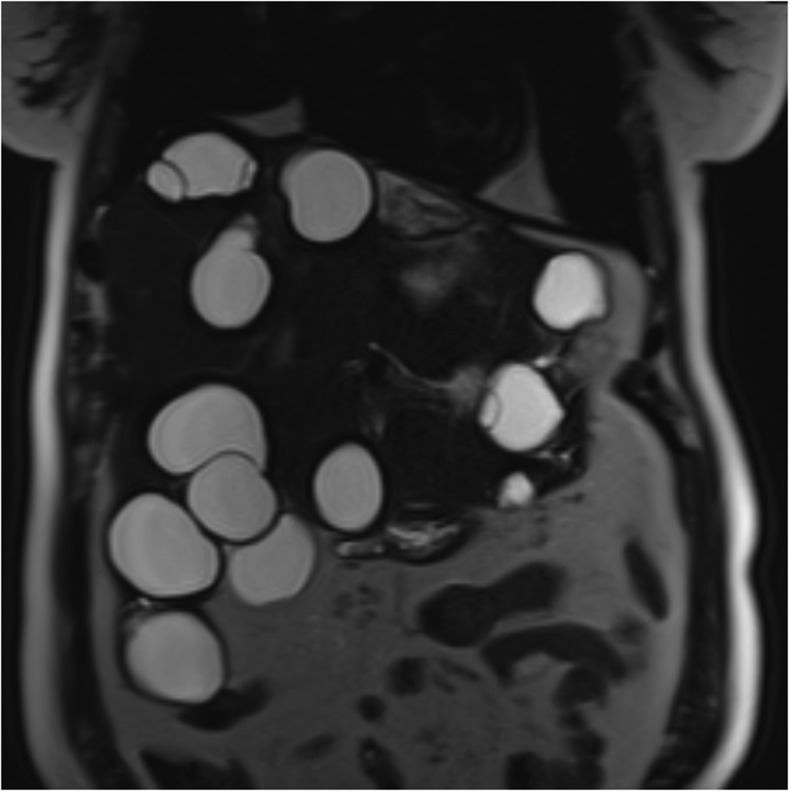
Coronal view of a T2-weighted magnetic resonance imaging (MRI) demonstrating multiple hydatid cysts of the liver.

A 53-year-old female refugee from northern Iraq presented to a hospital in Toronto, Canada, with clinical evidence of pyelonephritis. Ultrasonography incidentally identified multiple hepatic cysts. This prompted subsequent imaging with magnetic resonance imaging, with T2-weighted images highlighting multiple hepatic cysts throughout the liver parenchyma (Figure 1). Her pyelonephritis resolved with antimicrobial therapy and she was asymptomatic thereafter. Further computed tomography (CT) imaging studies demonstrated several small cysts in her lungs. *Echinococcus granulosus* serology was positive with a titer of 1:256. The patient was not deemed to be a candidate for surgical resection or a puncture–aspiration–injection–reaspiration (PAIR) procedure given her multiple hepatic and pulmonary cysts.^1^ She was treated with albendazole (400 mg orally, twice daily) for 1 year and remained completely asymptomatic. CT imaging at the completion of therapy revealed no new cysts and mild cyst regression, and CT imaging 9 months after completing the therapy demonstrated no further cyst growth. She remains asymptomatic 1 year after completing therapy. *Echinococcus* species commonly affect the liver and occasionally extrahepatic sites such as the lung.^2^ Cases may be treated medically if patients are not candidates for surgical or PAIR procedures.^1^
